# What Do We Know About the Energy Status and Diets of Pre-Professional and Professional Dancers: A Scoping Review †

**DOI:** 10.3390/nu16244293

**Published:** 2024-12-12

**Authors:** Alessandra Rigoli, Emily Dang, Victoria Michael, Janelle Gifford, Alyse Davies

**Affiliations:** 1 Discipline of Nutrition and Dietetics, Susan Wakil School of Nursing and Midwifery, Faculty of Medicine and Health, The University of Sydney, Camperdown, NSW 2006, Australia; 2 The Charles Perkins Centre, The University of Sydney, Camperdown, NSW 2006, Australia; 3 Discipline of Exercise and Sport Science, Sydney School of Health Sciences, Faculty of Medicine and Health, The University of Sydney, Camperdown, NSW 2006, Australia; 4 Sport and Physical Activity Research and Teaching Network (SPARTAN), The University of Sydney, Camperdown, NSW 2006, Australia

**Keywords:** dance, pre-professional, professional, ballet, contemporary, musical theatre, opera, performing arts, nutrition, diet, energy status

## Abstract

Background/Objectives: Dancers require adequate nutrition support for growth and development during the pre-professional stage, as well as to fuel classes and rehearsals and to enhance performance for both pre-professional and professional dancers. The aim of this study is to understand the energy status and diet of pre-professional and professional dancers in the genres of ballet, contemporary, musical theatre, and opera. Methods: Electronic databases (*n* = 9) and grey literature were searched for primary studies with no time limit. Screening and data extraction were completed by two reviewers. Results: Twelve studies were included for pre-professional (*n* = 7) and professional (*n* = 5) dancers. The genres identified were ballet (*n* = 11) and contemporary (*n* = 1), with no studies on musical theatre or opera. Studies on pre-professional ballet and contemporary dancers indicated a negative energy balance and low energy availability. Pre-professional ballet dancers had lower energy intakes than professional dancers. Professional dancers had lower BMI and body fat percentages. Macronutrients were mostly reported using the acceptable macronutrient distribution range for carbohydrates (38–56%E), protein (12–17%E), and total fat (26–42%E). Iron and calcium were the main micronutrients of concern. Conclusions: Accredited sports dietitians are recommended to support pre-professional and professional dancers to optimize their diet for health and performance. Further investigation is needed to quantify and assess dancers’ dietary intake using sports nutrition guidelines for reference.

## 1. Introduction

Dancers are not usually considered among the elite athlete population, despite having comparable energy demands in the volume and intensity of their training to many other sports [1]. Dance is an aesthetic sport that combines artistry and athleticism, with muscular strength, flexibility, cardiovascular endurance, and explosive power being characteristics of many genres [2]. Historically, it has been challenging to quantify specific energy requirements for dancers due to the large variability in intensity of dance styles, choreography, rehearsals, and class structures as well as performance demands and schedules [1,3,4]. Ballet classes are characterized by periods of higher intensity combinations, particularly at the end of class with allegro and coda, where jumps and turns are executed with explosive power [4]. Contemporary classes consist of continuous moderate intensity movement with fewer rest periods [4]. Musical theatre and opera productions may consist of intermittent bursts of moderate to high intensity activity, but the volume and intensity are highly dependent on the main genre and repertoire of the production. 

To achieve pre-professional status and pursue a career in dance, dancers train for several years from childhood to strive for technical precision. Pre-professional and professional dancers have long working days consisting of classes, conditioning, and repertoire five to seven days a week [5,6]. This often leaves very little time for rest or meal breaks, further increasing the challenges of maintaining adequate nutritional intake. This is of particular concern during pre-professional years when dancers are undergoing significant growth and development [7]. Once a show is in production, the schedule usually consists of matinee and evening performances, with up to eight shows per week for certain genres. Productions may also tour nationally or internationally, which can introduce additional barriers in achieving nutritional adequacy, such as food unfamiliarity or scarcity [8]. Meals may be catered for on double show days, which may not be conducive to performance as dietitians typically have no input.

The complexity of defining energy needs within the art form, coupled with aesthetic ideals, means that dancers may be unable to achieve nutritional adequacy to support their training and performance demands. The unique pressures of the craft to achieve certain physiques that are considered advantageous for career progression also place dancers at an increased risk of engaging in disordered eating behaviors and developing eating disorders [9]. In their systematic review and meta-analysis, Arcelus et al. (2014) found that dancers had a 2.5 times greater risk of having an eating disorder than non-dancers, with 12% of all dancers and 16% of ballet dancers having been diagnosed with an eating disorder [10]. Under-fueling is also prevalent in dancers; evidence suggests they consume 70–80% of their energy requirements [2] and micronutrient deficiencies are common [7,11]. High training volumes and restricted dietary intake, either intentional or unintentional, result in low energy availability (LEA) [12]. Prolonged (“problematic”) LEA leads to the development of relative energy deficiency in Sport (RED-S) [12], where decreased coordination, muscle strength, endurance, and training response, and increased fatigue and injury risk is observed [13]. This may result in career threatening injuries such as fractures due to depleted bone mineral density, impaired performance, disrupted menses, and deleterious endocrine and metabolic consequences that can persist beyond their career [12,13].

More recently, many dance and theatre companies have adopted a holistic approach to dancers’ health and wellbeing; however, dietitians are not usually actively included in these support networks and may be hired only as external consultants [14]. There are also no current consensus guidelines to provide a standard for recommendations for dancers and their sport-specific requirements. This study aims to scope the existing literature to understand the energy status and dietary intake of pre-professional and professional dancers. This review will provide the necessary groundwork for future research to quantify nutritional requirements in dance and increase dietetic involvement within the performing arts industry.

To place this research in the context of dance and nutrition, a positionality statement is important. Two members of the research team (A.R. and A.D.) have a dance background. A.R. has trained pre-professionally in contemporary, ballet, and other commercial dance genres and is a Provisional Sports Dietitian. A.D. has trained pre-professionally and performed professionally over a period of 17 years in ballet, musical theatre, and opera and is an Accredited Sports Dietitian. J.G. is a Fellow of Sports Dietitians Australia and has practice experience and theoretical knowledge on diet and nutrition for elite athletes. E.D. and V.M. were student dietitians with a strong interest in dance. 

## 2. Materials and Methods

### 2.1. Protocol and Registration

The scoping review was conducted following the Arksey and O’Malley framework [15]. Results were reported in accordance with the Joanna Briggs Institute ‘Updated Methodological Guidance for Scoping Reviews’ [16]. The protocol was registered on Open Science Platform (https://osf.io/5zksh/, accessed on 10 May 2023). Ethics approval was not required for this review. 

### 2.2. Inclusion 

#### 2.2.1. Participants

Participants had to consist of pre-professional and/or professional dancers, whose data could be extracted according to their specific level and genre of dance. Pre-professional dancers were defined as individuals studying full-time (at least 5 days or 35 h/week) at a dance or performing arts school. Pre-professional terms varied across the literature, including “professional schools”, “conservatory schools/dancers”, “vocational dancers”, and ‘full-time’. Professional dancers included those on a salary or contracted for a production for the genres of ballet, contemporary, musical theatre, or opera performers, with all other genres excluded.

#### 2.2.2. Concept

Studies that reported on energy status and dietary intake were included. Methods of measuring dietary intake, including quantitative, qualitative, and mixed methods, were included in this review. Studies that reported exclusively on nutrition knowledge, supplement use, alcohol intake, and disordered eating were excluded due to them not being reflective of dietary intake. 

#### 2.2.3. Context

This review included studies from any country. Published scientific literature and peer reviewed articles sourced from grey literature searches were included. 

### 2.3. Types of Sources

Primary studies that assessed the dietary intake of dancers, including any aspect of energy availability (EA), macro- and micronutrient intake, beverage consumption including alcohol, and disordered eating behaviors (e.g., skipping meals, dieting, avoiding eating), were included. Government reports, reviews and meta-analyses, protocols, conference abstracts, theses, websites, magazine or newspaper articles, blogs, and editorials were excluded. Relevant review references were screened to capture any additional studies. Studies were excluded if the full text was unavailable or if they were not in English. 

### 2.4. Search Strategy

A comprehensive search strategy was developed by one researcher (A.D.) and experienced librarian, using a combination of MeSH headings and keywords identified in titles and abstracts. The initial search was performed in MEDLINE to identify relevant MeSH headings and keywords, which were then modified for use in additional databases. No publication restrictions were applied due to the scarcity of existing published research and studies were limited to the English language. The full search was conducted in September 2023 using nine electronic databases (Medline, Embase, Central, Cinahl, SportDiscus, Premium Arts Collection, International Index to Performing Arts, and Ausport). A modified search using key terms was conducted on Google Scholar, with the first 200 results included. The search strategy for MEDLINE is presented in Supplementary Table S1. Weekly alerts were established in all databases. 

### 2.5. Selection Process

**Figure 1:**
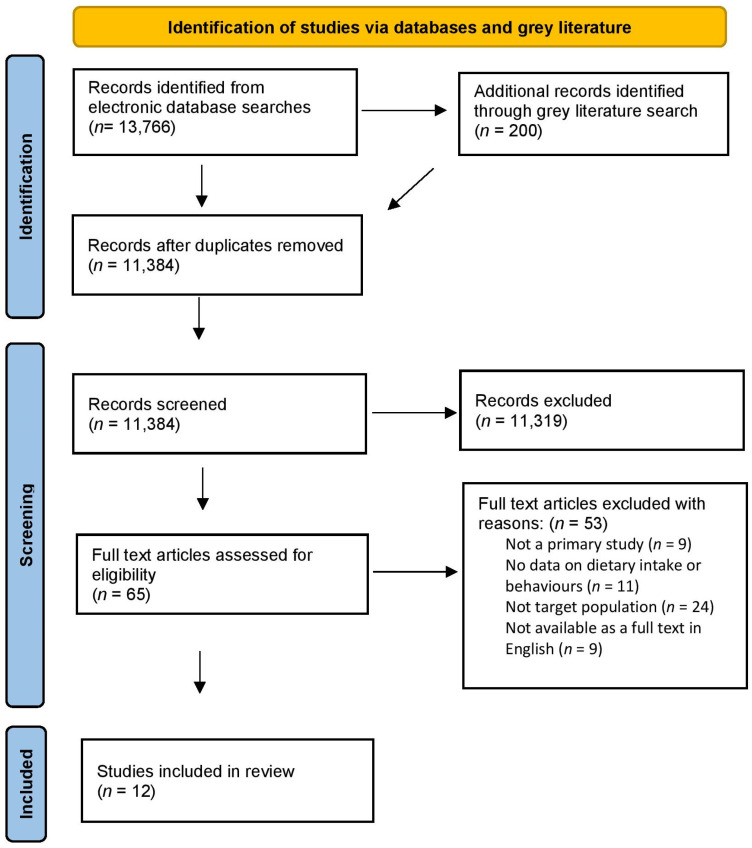
PRISMA Flow diagram of identifying eligible papers according to inclusion/exclusion criteria.

Publications that were identified through the full search strategy were imported into Endnote 20 citation management software (Clarivate Analytics, Philadelphia, PA, USA), and duplicates were removed. The title and abstracts were screened by two independent reviewers (E.D., V.M.) in Covidence (Veritas Health Innovation, Melbourne, Australia) against the review inclusion criteria. The studies that satisfied the inclusion criteria then underwent full text screening by two independent reviewers (E.D., V.M.). Conflicts during screening and extraction were discussed between the two reviewers and resolved by consensus in weekly meetings with the supervisory team (A.R., J.G., A.D.). The search results are presented in an adapted PRISMA flow diagram (Figure 1). 

### 2.6. Data Extraction and Charting

Data extraction followed a standardized data charting form based on an existing framework for scoping reviews [17] and was conducted by two independent reviewers (E.D., V.M.). This included study (first author, title, journal and year of publication, country of origin, study design, dietary assessment method, and number of days if applicable) and participant characteristics (age, size and sex composition of sample, genre of dance, pre-professional or professional status) as well as primary outcomes (energy intake (EI), energy expenditure (EE), body composition, macronutrient intake, micronutrients, food groups, and beverage consumption,) and secondary outcomes (nutrition knowledge, supplement use, disordered eating behaviors).

Only baseline data from a three-year longitudinal study was extracted for Amorim 2021 [18]. 

### 2.7. Synthesis of Results

The results were presented in both tabular and written form, with an accompanying narrative summary to describe the energy status and dietary intake among pre-professional and professional dancers.

## 3. Results

### 3.1. Search Results

Database and grey literature searching identified a total of 13,966 records. Following the removal of duplicates, 11,384 title and abstracts were screened and 11,319 were excluded. A full text review of 65 records was carried out to assess eligibility and 53 articles were excluded. A total of 12 papers were included in this review [18,19,20,21,22,23,24,25,26,27,28,29]. The PRISMA flow diagram details this selection process (see Figure 1).

### 3.2. Study Characteristics

The study characteristics are presented in Table 1. There were seven studies on pre-professional dancers [18,19,20,21,22,23,24] and five on professional dancers [25,26,27,28,29], with none reporting on both pre-professional and professional dancers. The age range of pre-professional dancers was 11 to 29 years old and professional dancers 17 to 42 years old. Eleven studies focused on ballet [18,19,20,21,22,23,25,26,27,28,29] and one on contemporary dancers [24], with no studies on musical theatre or opera. All studies were conducted in Western countries, with 50% based in the USA [20,21,25,27,28,29]. Publication dates ranged from 1985 to 2021. Only three studies included male participants, two of which were on professional ballet dancers [25,26] and one on pre-professional ballet dancers [19]. The studies on pre-professional dancers had larger sample sizes, ranging from 25 to 160 participants, whereas samples of professional dancers ranged from 10 to 22 participants. 

### 3.3. Energy Status

The energy status of pre-professional and professional dancers are presented in Table 2. Five studies reported on pre-professional dancers’ energy status, with four on ballet dancers [19,20,22,23] and one on contemporary dancers [24]. Five studies reported on professional ballet dancers [25,26,27,28,29]. Energy status was recorded as EI, EE, and EA. 

**Table 1:** Study characteristics of pre-professional and professional dancers.

First Author, Year, Ref	Country	Population	Genre	Study Design	Sample Size, Gender, Ethnicity	Age Range in Years, Age in Years (Mean ± SD)
Abraham, 1996, [18]	Australia	Pre-professional	Ballet	Cross-sectional (with comparison group)	*n* = 60F	NR
Amorim, 2021, [19]	Portugal	Pre-professional	Ballet	Longitudinal	*n* = 101 63F, 38M White European-Caucasian	F: 12.8 ± 2.2 M: 12.7± 2.2
Benson, 1985, [20]	USA	Pre-professional (six professional schools)	Ballet	Cross-sectional	*n* = 92F	12–17 y 14.6
Braisted, 1985, [21]	USA	Pre-professional (Nationally recognized professional dance school)	Ballet	Cross-sectional (with comparison group)	*n* = 45F Caucasian	12–21 y 16.1 ± 2.3
Civil, 2018, [22]	Scotland	Pre-professional 1st year of study (*n* = 8) 2nd year of study (*n* = 11) 3rd year of study (*n* = 4)	Ballet	Cross-sectional	*n* = 20F Caucasian	18.1 ± 1.1
Dotti, 2002, [23]	Italy	Pre-professional	Ballet	Cross-sectional	*n* = 160F Group 1: Girls (*n* = 75) Group 2: Adolescents (*n* = 36) Group 3: Young Adults (*n* = 30) Group 4: Adults (*n* = 19)	11–29 y Group 1: 11–14 12.6 ± 0.8 Group 2: 15–18 16.18 ± 1.1 Group 3: 19–22 20.5 ± 1.1 Group 4: 23–29 24.5 ± 1.9
Brown, 2017, [24]	UK	Pre-professional	Contemporary	Cross-sectional	*n* = 25F	21.0 ± 2.0
Cohen, 1985, [25]	USA	Professional Principal (*n* = 3), Soloists (*n* = 4) Corps de ballet (*n* = 15)	Ballet	Cross-sectional	*n* = 22 12F, 10M	17–31 y F: 24.4 ± 3.8 M: 26.0 ± 3.6
Da Silva, 2016, [26]	Brazil	Professional	Ballet	Cross-sectional	*n* = 10 5F, 5M	20–42 y F: 29.6 ± 8.6 M: 26.8 ± 8.8
Doyle Lucas, 2010, [27]	USA	Professional (two national professional ballet Companies)	Ballet	Cross-sectional (with matched controls)	*n* = 15F	18–35 y 24.3 ± 1.3
Hamilton, 1986, [28]	USA	Professional (four companies, completed ≥8 years of ballet training before entering their companies)	Ballet	Cross-sectional	*n* = 19F	24.9 20–36
Hoch, 2011, [29]	USA	Professional (one company, 17.0 ± 4.5 mean years of elite dancing)	Ballet	Cross-sectional	*n* = 22F White (*n* = 20); Asian (*n* = 1); Hispanic (*n* = 1)	23.2 ± 4.7 18–35

All values have been rounded to the nearest decimal place. F, Female; M, Male; NR, Not Reported.

**Table 2:** The energy status, anthropometry and body composition of pre-professional and professional dancers, including energy intake.

First Author, Year, Ref	Level, Genre	Dietary Assessment Method	Energy Intake (Mean kJ/Day ± SD)	Energy Expenditure Assessment Method	Energy Expenditure (Mean kJ/Day ± SD)	Energy Balance (Mean kJ/Day ± SD)	Energy Availability (Mean kJ/kg FFM/Day ± SD)	Anthropometric Data (Mean ± SD): Height (cm), Weight (kg), BMI (kg/m^2^), Body Fat (BF%), Fat-Free Mass (FFM) (kg)
Amorim, 2021, [19] ^a^	Pre-professional, Ballet	3-day validated food diary (two school days, one WE day)	F = 7215 ± 2125 M = 6691 ± 1941	SenseWear^TM^ accelerometer for 7 consecutive days	NR	NR	F = 196 ± 96 ^c^ M = 151 ± 76 ^c^	Stadiometer, electronic scale: F: Height = 151.7 ± 10.5 Weight = 39.3 ± 9.0 M: Height = 156.6 ± 15.8 Weight = 44.6 ± 13.5
Benson, 1985, [20] ^a^	Pre-professional, Ballet	3-day diet record (two WDs, one WE day)	F = 7909 48.5% consumed <7531 kJ/day. 28.9% consumed <6276 kJ/day. 10.8% consumed <5021 kJ/day.	NR	NR	NR	NR	Height = 160 Weight = 47 ^g^
Civil, 2018, [22] ^a^	Pre-professional, Ballet	7-day weighed food diary (WD—average of 5 day; WE—average of 2 days), cross- referenced with 24-hr recall in order to add may missing data or clarify ambiguous information.	7-day period = 8222 ± 1665 WD = 8276 ± 1904 WE = 8795 ± 2536	Tri-axial ActiGraph GT3X+ accelerometer for 7 consecutive days and activity log	7-day period = 9703 ± 925 WD = 10054 ± 937 WE = 8820 ± 1096	7-day period = −1298 ± 1556 WD = −1778 ± 1950 * WE = −25 ± 2117 *	7-day period = 165 ± 45 WD = 158 ± 55 ^c^ WE = 185 ± 54 ^c^	Stadiometer, electronic scale: Height = 169 ± 54 Weight = 54 ± 6 BMI = 19 ± 2 BF% ^e^ = 23 ± 3 FFM ^e^ = 43 ± 4
Dott, 2002 [23] ^a^	Pre-professional, Ballet	24-h and 3- day dietary recall	11–14 y = 7374 ± 1478 1518 y = 6531 ± 2078 19–22 y = 6379 ± 1615 23–29 y = 6283 ± 1581	NR	NR	NR	NR	11–14 y: Height = 150 ± 6 Weight = 39 ± 5 BMI = 17 ± 2 15–18 y: Height = 159 ± 4 Weight = 47 ± 5 BMI = 19 ± 1 19–22 y: Height = 160 ± 2 Weight = 52 ± 5 BMI = 20 ± 1 23–29 y: Height = 159 ± 5 Weight = 52 ± 6 BMI = 20 ± 2
Brown. 2017, [24] ^a^	Pre-professional, Contemporary	7-day weighed food diary, cross- referenced with a 24-h recall to clarify ambiguous information and complete diary entries with missing data	7-day period = 10,159 ± 1916 WD = 9611 ± 2059 * WE = 11,531 ±2799 *	Tri-axial ActiGraph GT3X+ accelerometer for 7 days	7-day period = 11,648 ± 2381 WD = 11376 ±1703 WE = 11,016 ±2402	7-day period = -1490 ± 2795 WD = −1766 ± 2146 * WE = −515 ± 4213 *	7-day period = 109 ± 54 WD = 100 ± 42 * ^c^ WE = 151 ± 88 * ^c^	Height = 167 ± 6 Weight = 63 ± 7 BMI = 23 ± 2 BF% ^d^ = 28 ± 3 FFM = 46 ± 4
Cohen, 1985, [25] ^a^	Professional, Ballet	6-day estimated food diary, cross- referenced with an interview	F = 7000 ± 1883 M = 12,414 ± 2791	NR	NR	NR	NR	Electronic scale F: Height = 166 ± 4 Weight = 48 ± 3 BF% ^d^ = 13 ± 2 M: Height = 180 ± 5 Weight = 70 ± 6 BF% ^d^ = 8 ± 1
Da Silva, 2016, [26] ^a^	Professional, Ballet	24-hr recall	F = 9351 ± 1668 M = 10,286 ± 1507	NR	NR	NR	NR	F: Weight = 56 ± 2 BMI = 21 ± 0 BF% ^d^ = 17 ± 2 M: Weight = 75 ± 9 BMI = 24 ± 3 BF% ^d^ = 10 ± 3
Doyle- Lucas, 2010, [27] ^a^	Professional, Ballet	4-day estimated dietary record	6514 ± 372	NR	NR	NR	16 ± 9 ^b, c^	Stadiometer, electronic scales Height = 166 ± 1 Weight = 52 ± 1 BMI = 19 ± 0 BF% ^e^ = 16 ± 1 FFM ^e^ = 44 ± 1
Hamilton, 1986, [28] ^a^	Professional, Ballet	24-h food record of a typical performance week	7925 ± 3580	NR	NR	NR	NR	Height = 167 ^f^ Weight = 51 ^g^
Hoch, 2011, [29]	Professional, Ballet	3-day estimated food record (2 WDs, one WE day) during the training season	NR	Tri-axial Actigraph GT1M accelerometer continuously for three days	NR	−2292 ± 1506	77% (*n* = 17) had low/negative EA	Height = 165 ± 7 Weight = 52 ± 5 ^g^ BMI = 19 ± 1 BF% ^e^ = 17 ± 5

^a^ all values for energy intake, expenditure and balance were converted from kcal to kJ using a conversion factor of 4.184 and rounded to the nearest whole number. ^b^ energy availability was updated using the mean variables for kcal and kg. ^c^ all values for EA were converted from kcal/kg FFM/day to kJ/kg FFM/day using a conversion factor of 4.184 and rounded to the nearest whole number. ^d^ Skinfold thickness used to measure body fat percentage. ^e^ DEXA used to measure anthropometric data. ^f^ Values converted to centimeters from inches. ^g^ Values converted to kilograms from pounds. * significant difference reported (*p* < 0.05). NR, Not Reported; All values have been rounded to the nearest whole number.

#### 3.3.1. Energy Intake

Ten studies reported on EI [19,20,22,23,24,25,26,27,28,29], with five collecting dietary data using a food dairy/diet record [19,20,27,28,29], two using recall methods [23,26], two using diet records cross referenced with 24-h recall [22,24], and one cross referencing diet records with an interview [25]. Professional ballet dancers generally had higher EI (6514–12,414 kJ/day) than their pre-professional counterparts (5021–11,531 kJ/day). Pre-professional contemporary dancers had higher EI [24] than pre-professional ballet dancers. EI was significantly higher on weekends compared to weekdays for pre-professional contemporary dancers (*p* = 0.002) [24]. 

#### 3.3.2. Energy Expenditure

Energy Expenditure was measured in four studies using an accelerometer [19,22,24,29] and reported in one study each on pre-professional ballet and contemporary dancers. Energy Expenditure was not reported in professional dancers. Pre-professional contemporary dancers had higher EE on weekdays and weekends compared to pre-professional ballet dancers [22,24]. Energy Expenditure was reduced on the weekend in pre-professional ballet dancers [22]. 

#### 3.3.3. Energy Balance

Energy balance was reported in three studies, all reporting a negative energy balance [22,24,29]. Energy deficits were lower on weekdays compared to weekends in both pre-professional ballet and contemporary dancers [22,24]. 

#### 3.3.4. Energy Availability

Energy Availability was reported in five studies, all of which reported a low or negative EA [19,22,24,27,29]. Professional ballet dancers had lower EA [27,29] in comparison to pre-professional ballet and contemporary dancers [19,22,24]. Male pre-professional ballet dancers had an EA of 151 kJ/kg FFM/day and female ballet dancers’ EA ranged from 158 to 196 kJ/kg FFM/day [19,22]. Pre-professional ballet and contemporary dancers both exhibited improved EA on weekends, with ballet dancers recording an EA of 158 kJ/kg FFM/day on weekdays and 185 kJ/kg FFM/day on weekends [22] and contemporary dancers reporting an EA of 100 kJ/kg FFM/day on weekdays and 151 kJ/kg FFM/day on weekends [24]. The EA of professional ballet dancers reported in one study was as low as 16 kJ/kg FFM/day [27] and another reported 77% of dancers having low or negative EA [29]. 

#### 3.3.5. Anthropometry and Body Composition

Anthropometry was assessed using a stadiometer [19,22,26] and electronic scales [19,22,25,26]. Body composition was assessed with either equations using skinfold measurements [24,25,26] or dual energy x-ray absorptiometry [22,27,29]. Body Mass Index (BMI) and body fat percentage were lower in professional ballet dancers [25,26,27,29] compared to pre-professional ballet [22,23] and contemporary dancers [24].

### 3.4. Macronutrient Intake 

Table 3 outlines the data on dancers’ macronutrient intake from nine studies [19,20,22,23,24,25,26,27,28]. All reported on carbohydrate and fat and eight on protein [20,22,23,24,25,26,27,28]. Only two studies used grams per kilogram of bodyweight per day for carbohydrates (4.8–5.4), protein (1.2–1.4), and fat (1.3–1.8) [22,24].

**Table 3:** Macronutrient composition and beverage consumption of pre-professional and professional dancers.

First Author, Year, Ref	Level, Genre	Average Intake of CHO (g/day ± SD; g/kg BW/day ± SD); %E	Fiber (g/gay)	Average Intake of Protein (g/day ± SD; g/kg BW/day ± SD); %E	Average Intake of Fat (g/day ± SD; g/kg BW/day ± SD);%E	Beverages
Amorim, 2021, [19]	Pre-professional, Ballet	F = 225 ± 70 g/day M = 211 ± 69 g/day *	NR	NR	F = 59 ± 84 g/day * M = 55 ± 19 g/day *	NR
Benson, 1985, [20]	Pre-professional, Ballet	236 ± 101 g/day; 50%E	NR	72 ± 24 g/day; 16%E	75 ± 37 g/day; 35%E PUFA/SFA: 2:3	NR
Braisted, 1985, [21]	Pre-professional, Ballet	NR	NR	NR	NR	One participant considered juice or water an aid to performance.
Civil, 2018, [22]	Pre-professional, Ballet	7-day period: 263 ± 55 g/day; 4.9 ± 1.1 g/kg BW/day; 53 ± 4%E WD = 263 ± 66 g/day; 4.9 ± 1.3 g/kg BW/day; 54 ± 4%E WE = 261 ± 64 g/day; 4.8 ± 1.1 g/kg BW/day; 53 ± 6%E	7-day period: 30 ± 12 WD = 33 ± 13 WE = 26 ± 11	7-day period: 66 ± 12 g/day; 1.2 ± 0.2 g/kg BW/day; 14 ± 3%E WD = 66 ± 15 g/day; 1.2 ± 0.3 g/kg BW/day; 14 ± 3%E WE = 66 ± 18 g/day; 1.2 ± 0.3 g/kg BW/day; 14 ± 3%E	7-day period: 75 ± 22 g/day; 1.4 ± 0.4 g/kg BW/day; 33 ± 5%E WD = 73 ± 23 g/day; 1.4 ± 0.4 g/kg BW/day; 33 ± 5%E WE = 78 ± 32 g/day; 1.4 ± 0.6 g/kg BW/day; 33 ± 7%E	Fluid (mL/d): 7-day period = 1649 ± 488 WD = 1768 ± 521 * WE = 1350 ± 472 * WE = 1350 ± 472 * Alcohol ((*n* = 4): 7-day period = 8 ± 21 g/day no significant difference between WD and WE.
Dotti, 2002, [23]	Pre-professional, Ballet	11–14 y: 209 ± 48 g/day; 51%E 15–18 y: 196 ± 76 g/day; 54%E 19–22 y: 192 ± 70 g/day; 55%E 23–29 y: 179 ± 48 g/day; 52%E	NR	11–14 y: 68 ± 16 g/day; 15%E 15–18 y: 56 ± 20 g/day; 14%E 19–22 y: 53 ± 12 g/day; 14%E 23–29 y = 58 ± 16 g/day; 15%E	11–14 y: 68 ± 15 g/day; 34%E 15–18 y: 56 ± 20 g/day; 32%E 19–22 y: 53 ± 16 g/day; 31%E 23–29 y = 56 ± 19 g/day; 33%E	NR
Brown, 2017, [24]	Pre-professional, Contemporary	7-day period: 313 ± 58 g/day; 5.0 ± 1.0 g/kg BW/day; 52 ± 7%E WD = 304 ± 57 g/day; 4.8 ± 0.8 g/kg BW/day; 54 ± 7%E * WE = 335 ± 97 g/day; 5.4 ± 1.7 g/kg BW/day; 49 ± 8%E *	NR	7-day period: 81 ± 15 g/day; 1.3 ± 0.3 g/kg BW/day; 13 ± 2%E WD = 79 ± 17 g/day; 1.3 ± 0.3 g/kg BW/day; 14 ± 2%E WE = 85 ± 22 g/day; 1.4 ± 0.5 g/kg BW/day; 13 ± 3%E	7-day period: 92 ± 30 g/day;1.5 ± 0.4 g/kg BW/day; 34 ± 5%E WD = 85 ± 33 g/day; 1.3 ± 0.5 g/kg BW/day; 32 ± 6%E * WE = 110 ± 33 g/day; 1.8 ± 0.6 g/kg BW/day; 36 ± 6%E *	Alcohol 7-day period: 9 ± 13 g/day: 0.2 ± 0.2 g/kg BW/day; 3 ± 4%E WD = 5 ± 14 g/day; 0.1 ± 0.3 g/kg BW/day; 2 ± 5%E * WE = 20 ± 22 g/day; 0.3 ± 0.4 g/kg BW/day; 5 ± 5%E *
Cohen, 1985, [25]	Professional, Ballet	F = 207 ± 71 g/day; 50 ± 14%E M = 300 ± 86 g/day; 38 ± 8%E	NR	F = 59 ± 19 g/day; 14 ± 2%E M = 122 ± 24 g/day; 17 ± 2%E	F = 71 ± 29 g/day; 38 ± 10%E M = 140 ± 44 g/day; 42 ± 7%E	NR
Da Silva, 2016, [26]	Professional, Ballet	F: 51 ± 9%E M: 56 ± 3%E	F = 12 ± 3 M = 13 ± 7	F: 12 ± 2%E M: 15 ± 3%E	F: 37 ± 8%E; SFA 9 ± 5%E M: 29 ± 3%E; SFA 8 ± 4%E	NR
Doyle-Lucas, 2010, [27]	Professional, Ballet	56 ± 3%E	NR	17 ± 1%E	26 ± 2%E	Alcohol 4 ± 1%E
Hamilton, 1986, [28]	Professional, Ballet	281 ± 274 g/day	NR	88 ± 50 g/day	405 ± 380 g/day	NR

* significant difference reported (*p* < 0.05). All values have been rounded to the nearest whole number. F, Female; M, male; WD, Weekday; WE, Weekend day; NR, Not Reported; SFA, saturated fatty acids intake; y, years; d, days; %E, percent energy.

#### 3.4.1. Carbohydrates

Seven studies reported %E from carbohydrates ranging between 49–56%E [19,20,23,24,26,27], with the exception of one outlier of 38%E for professional male ballet dancers [25]. Pre-professional contemporary dancers had higher carbohydrate intakes compared to both pre-professional and professional ballet dancers. Contemporary dancers consumed 313 g carbohydrates/day, averaging 300 g carbohydrates/day on weekdays and 335 g/day on weekends [24]. Pre-professional ballet dancers’ carbohydrate intake ranged from 179 to 263 g/day [19,20,22,23]. There was little difference between carbohydrate intake on the weekend compared to weekdays in pre-professional ballet dancers [22]. Professional ballet dancers consumed between 207 and 300 g of carbohydrate/day [25,28].

#### 3.4.2. Fiber

Fiber was reported in two studies as g/day [22,26]. Pre-professional ballet dancers consumed 33 g fiber/day on weekdays and 6 g/day on weekends [22]. Fiber intake in professional ballet dancers was 12 g/day for females and 13 g/day for males [26]. 

#### 3.4.3. Protein

Six studies reported on the %E from protein, ranging between 12 and 17%E [20,23,24,25,26,27]. Protein intake ranged from 53 to 122 g/day [20,22,23,24,25,28]. There was little difference in protein intake between pre-professional and professional dancers. Pre-professional contemporary dancers had higher protein intakes than pre-professional ballet dancers on both weekdays and weekends. Across 7 days, contemporary dancers consumed 81 g/day of protein [24] compared to 66 g/day in ballet dancers [22]. Contemporary dancers consumed 79 g/day of protein on weekdays and 85 g/day on weekends [24] compared to 66 g/day on both weekdays and weekends in ballet dancers [22]. Male professional ballet dancers had the highest protein intakes of 122 g/day [25]. 

#### 3.4.4. Fat

Three studies reported on %E from fat ranging between 26–42%E [20,24,26]. Average fat intake ranged from 53 to 405 g/day [19,20,22,23,24,25,28]. Professional ballet dancers had the highest mean recorded intake at 405 g/day [28]. Pre-professional contemporary dancers had higher fat intakes (85–110 g/day) than pre-professional ballet dancers (53–78 g/day) [19,20,22,23,24]. Two studies reported on saturated fat intake [20,26]. Female professional ballet dancers consumed 8%E of saturated fat and male professional ballet dancers consumed 9%E of saturated fat [26].

### 3.5. Micronutrient Intake

Six studies reported on the iron intake of ballet dancers [20,23,25,27,28,29]. Average iron consumption ranged between 7 and 13 mg per day for pre-professional dancers [20,23] and 12 and 42 mg per day for professional dancers [25,27,28,29]. Eight studies reported dietary calcium intake, ranging between 527 and 933 mg per day for pre-professional [19,20,23] and 463 and 1031 mg for professional dancers [25,26,27,28,29]. Male dancers had higher dietary iron and calcium intake than females [19,25]. Hoch et al. (2011) reported iron deficiency in 46% of professional ballet dancers and calcium deficiency in 59% [29]. Other micronutrients studies included vitamin A (*n* = 4) [20,25,26,28], potassium (*n* = 3), B vitamins (*n* = 3), vitamin C (*n* = 3) [20,25,28], magnesium (*n* = 2), and phosphorus (*n* = 2) [20,26]. Benson et al. (1985) was the only study reporting on sodium, zinc, cholesterol, and vitamin E [20]. 

### 3.6. Supplement Use

Supplement use was reported for four studies [20,21,22,25]. Bensen et al. (1985) reported that 60% of pre-professional ballet dancers routinely took vitamin or mineral supplements but only 7% consumed supplements in an adequate dosage to improve any nutritional deficiencies present [20]. Between 50–60% of both pre-professional [21,22] and professional ballet dancers [25] took large doses of B-, C-, and fat-soluble vitamins without evident deficiencies. 

### 3.7. Food Groups and Beverages

Two studies reported on fluid intake, including water, alcohol, and juice [21,22]. In one study, mean daily fluid intake was 1768 mL in pre-professional ballet dancers on weekdays and decreased to 1350 mL on the weekend [22]. Two studies reported on alcohol intake [22,24]. Pre-professional ballet dancers’ alcohol intake did not significantly differ across the week, averaging 8 g/day [22], whereas pre-professional contemporary dancers’ alcohol intake increased from 5 g/day during the week to 20 g/day on the weekend [24].

### 3.8. Dietary Behaviours and Disordered Eating

Six studies assessed the eating habits and behaviors of dancers, one through semi-structured interviews [23] and five through questionnaires [18,21,22,25,28]. Dietary restriction was prevalent in all these studies [18,21,22,23,25,28]. Pre-professional ballet dancers (49%) indicated that they dieted ‘often’ or ‘always’, with 55% dieting for a week or longer [21]. Nineteen percent fasted ‘sometimes’ or ‘often’, with 45% of pre-professional ballet dancers fasting for longer than two days and 52% of fasters doing so as a weight loss strategy [21]. Fasting was unintentional in professional ballet dancers, attributed to long rehearsal days and performances without scheduled meal breaks [25]. Little nutritional variety and unbalanced meals were also observed in Cohen et al. (1985) [25]. Professional ballet dancers consumed the same or similar foods daily, and vegetables, grains, protein foods, and dairy were only eaten together occasionally [25]. Avoidance of red meat and dairy was common amongst pre-professional [22] and professional dancers [25,28]. 

Seventy-seven percent of pre-professional ballet dancers experienced difficulty controlling their eating and felt this to be a major problem, with half reporting that difficulty controlling their weight interfered ‘a lot’ with daily living [18]. Fifty-eight percent were preoccupied with thoughts of weight, food, or eating [18]. Common diet modifications of pre-professional ballet dancers included eating more fruit, vegetables, and salads (49%), reducing the overall volume of food eaten (29%), having a more balanced diet (22%), and eating less starch (17%) [21]. Non-dietary methods for weight loss or prevention of weight gain by pre-professional ballet dancers included use of laxatives (27%) or self-induced vomiting (20%) [18]. Fifteen percent of pre-professional ballet dancers ‘usually’ or ‘sometimes’ take above the recommended dose of laxatives, meanwhile diuretics have been trialed but are not preferred [18]. Binge eating behaviors were also prevalent in 32% pre-professional dancers [18], with episodes of bingeing occurring significantly more frequently in dancers (87%) than non-dancers (49%; *p* < 0.005) [21]. 

### 3.9. Nutrition Knowledge

One study reported on nutrition knowledge, finding that knowledge of performance nutrition varied [21]. Braisted et al. (1985) reported that 36% of pre-professional ballet dancers did not believe any foods aided performance, with others citing ‘protein’ (23%), ‘vegetables/salad’ (23%), ‘fruit’ (28%), and forms of complex carbohydrates (13%) [21]. Thirty-one percent of pre-professional ballet dancers did not believe any nutrients aided performance. Protein and Vitamin C were thought to aid performance, with few citing Vitamin A, B, and iron and calcium as important performance aids [21]. High fat and sugar foods were believed to hinder performance, with 21% believing no foods to be a hindrance [21]. Pre-professional ballet dancers sourced their nutrition knowledge from other athletes (75%), friends (61%), parents (52%), and teen magazines (48%), with only 5% reporting a nutritionist [21].

## 4. Discussion

While acknowledging the possibility of underreporting of dietary intake, it appears that dancers at both the pre-professional and professional level are inadequately fueling for the demands of their sport and are at risk of nutrition and energy deficiencies that may have deleterious consequences on their health and performance. This scoping review highlights limited literature on the energy status and diet of dancers, particularly across a broader range of genres. There is an imperative need for education by dietitians in the dance sphere to support adequate fueling and prevent negative outcomes associated with energy and nutrient deficiencies. 

### 4.1. Energy Balance 

Pre-professional ballet dancers reported the lowest EI. There was a large variability in EI observed in all groups, which can be attributed to a variety of factors that dancers are influenced by in their training. Dieting and restrictive eating behaviors were prevalent amongst both pre-professional and professional dancers [18,21,22,23,28]; this is likely a product of the stressful environments they are subject to. There is an element of competitiveness in dance based on aesthetic ideals, where dancers may manipulate their body to gain an advantage in securing roles and achieving success in their careers [9]. This is reflected in lower BMI and BF% of professional ballet dancers compared to pre-professional ballet and contemporary dancers [22,23,24,25,26,27,29], perhaps as a competitive edge to remain employable [9]. In the studio environment, wearing tight clothing such as leotards with pale pink tights and standing in front of mirrors for many hours can exacerbate fixation on bodies [9,30]. Coupled with the thin ideal upheld by instructors, this increases the risk of body dysmorphia and drives the pursuit of thinness in dancers [9,30]. Long rehearsal days with limited meal breaks also hinders the achievement of adequate EI [25]. Adolescents are more susceptible to body-related stressors and external sources of validation in this period of rapid physiological change [31], both intensified with the unique pressures of the dance environment. Dancers also have a much higher risk of developing eating disorders than non-dancers [10], attributed to the stressors faced in their craft. This further limits their capacity to achieve adequate EI to appropriately mitigate onset of LEA and RED-S. 

### 4.2. Energy Status

EA was found to be as low as 16 kJ/kg fat free mass (FFM)/day in professional ballet dancers [27]. EA defines the remaining energy after deducting exercise EE from EI that is available to sustain physiological function [12,13]. Optimal physiological function occurs at an EA of at least 188 kJ/kg FFM/day (45 kcal/kg FFM/day), especially in adolescents [32]. Disturbances to endocrine and metabolic parameters are observed at and below 126 kJ/kg FFM/day (30 kcal/kg FFM/day). Although only two studies fell below the threshold of LEA in pre-professional contemporary dancers (100 kJ/kg FFM/day) [24] and professional ballet dancers [27], reduced EA was observed in all dancers where reported [19,22,24,27,29]. Suboptimal performance and health consequences may be seen where EA is less than the ideal 188 kJ/kg FFM/day, thus highlighting the importance of addressing adequate EI to match EE in both professional and pre-professional dancers to prevent complications of LEA and subsequent RED-S, including amenorrhea [2,12,22,25,32,33,34] and low bone density [12,19,32,33,34,35], which are pervasive in these populations [36], as well a range of other health issues such as mental health issues, impaired gastrointestinal function, and sleep disturbances [12]. Male students, while underrepresented in our sample, are also susceptible to LEA, disordered eating, and eating disorders, although are generally underdiagnosed [37]. Preliminary research suggests that male athletes can sustain a lower EA before exhibiting physiological disturbances [12]; however, LEA compromises stamina, muscular strength, and bone and joint integrity, increasing injury risk for themselves and their female dance partners. Both pre-professional ballet and contemporary dancers exhibited improved energy balance on the weekends; however, contemporary dancers had stable EE on weekends [22,24]. Contemporary dancers may also face less pressures of aesthetic ideals than ballet dancers, as their clothing is usually looser fitting to accompany the style of the genre, and “athletic” bodies are believed to be more acceptable [9]. The variations of EI, EE, and EA between different genres merits further research into other dance styles to appropriately quantify energy needs to ensure nutritional adequacy, and to investigate the unique pressures experienced across genres and how they interact with EI. Investigating weekday and weekend differences in energy status parameters can assist in gaining a better understanding of the workload and adequate fueling required to support this. 

### 4.3. Macronutrients: Carbohydrates

Macronutrient intake was consistent across populations and genres. Carbohydrates typically accounted for 49–56% of total EI [19,20,23,24,26,27], falling within the defined acceptable range of 46–65% for the general population [38]. In athletes, carbohydrate intakes between 8 and 12 g/kg BW/day are recommended for both adolescents and adults training for at least 4–5 h/day at moderate to high intensities [7,39,40]. However, only two papers reported macronutrient intake in g/kg BW/day, limiting interpretation of dietary data as they may not be applicable to dancers within the athlete population. Neither pre-professional ballet dancers nor contemporary dancers met this recommendation when reported, with intakes of 4.8–4.9 g/kg BW/day [22] and 4.5–5.4 g/kg BW/day [24], respectively. Adequate carbohydrate intake is necessary to provide fuel for the central nervous system and lean tissues, and fluctuating blood glucose levels resulting from reduced carbohydrate availability have been linked with early fatigue and impaired cognition [41], which increases risk of injuries common in dancers such as foot and ankle injuries [42]. However, food high in carbohydrates is commonly avoided in dancers’ diets or recommended to be excluded by teachers [34].

### 4.4. Macronutrients: Protein

Pre-professional dancers consumed the lower end of recommended protein intakes of 1.3–1.8 g/kg BW/day for adolescent athletes [43], with ballet dancers averaging 1.2 g/kg BW/day [22] and contemporary dancers 1.3 g/kg BW/day [24]. No papers reported protein in g/kg BW/day for professional ballet dancers; however, intake as %EI was comparable to their pre-professional counterparts. Adequate protein intake is required for muscle growth and repair and production of hormones and enzymes to regulate normal physiological functioning, and are a secondary fuel source when glucose is scarce [41]. Low protein intake may be attributed to the common exclusion of dairy and red meat amongst dancers [22,25,28], which also explains the low iron and calcium intakes observed. 

### 4.5. Macronutrients: Fat 

Dietary fat intake accounted for 35% EI, despite the belief that high fat foods hindered performance [21] and documented avoidance of foods perceived to be high in fat, such as dairy products [23]. Higher %E from fat may be observed due to lower overall EI and avoidance of carbohydrate foods, thus leading to higher EI from fats. Professional ballet dancers often have 12-h training days without allocated lunch breaks [23], thus reliance on convenience foods may be a contributing factor, supported by their relatively high saturated fat intake [21]. Misconceptions surrounding sources of fats has been previously reported in Irish dancers, who had a 39% success rate for correctly identifying a food as high or low in fat [44]. It is plausible that ballet and contemporary dancers are unable to identify sources of fats and may unintentionally be consuming these in larger amounts. Alternatively, with the lower EI, the percentage of fat may be misleading in that with a higher EI and the same amount of fat, the percentage will be lower.

### 4.6. Micronutrients at Risk

Hoch et al. (2011) reported almost half of their sample being iron deficient and almost two-thirds calcium deficient [29]. All five papers [20,23,26,27,28] reporting numerical iron intakes met the EAR of 6 mg/day for those under 18 years and 8 mg/day for those aged 19–30. However, a 30% increase in iron requirements is usually recommended [45] for those performing high volume exercise to account for increased foot-strike hemolysis. The recommended daily intake of iron is 11 mg/day for males and 15 mg for females 14–18 years of age, and 8 mg/day for males and 15 mg/day for females aged 19–30 [46]. The four papers with female-only participants [20,23,26,27,28] narrowly achieved their respective requirements. Cohen et al. (1985) demonstrated a large disparity between the female and male iron intakes, consistent with the finding that males are less likely to exclude red meat [25,47]. Elevated requirements and suboptimal iron intake increase the risk of menstrual dysfunction, early fatigue, and decreased aerobic capacity in female dancers. Only two professional ballet samples [25,27] met their EAR of 840 mg calcium/day and no pre-professionals met their EAR of 1050 mg/day. Intense training loads and LEA triggers endocrine mediated bone resorption, which can result in low bone mineral density without sufficient calcium intake to counteract this process. With peak bone mass established in adolescence, it is concerning that 19% of injuries in pre-professional dancers were stress fractures [35]. Adequate intakes of carbohydrates, protein, and calcium supports optimal recovery and would reduce the risk of overuse injuries, which is critical in a weight-bearing sport like dance [42].

### 4.7. Supplements

Despite suboptimal micronutrient intakes, supplements appear to be underutilized by dancers. One in two ballet dancers regularly took vitamin or mineral supplements where no deficiency was evident [21,22,23]. Megadoses of B complex or Vitamin C were commonly consumed, consistent with the belief that vitamin C aided performance [21]. Where nutritional deficiencies were present, only 7% were consuming supplements in an adequate dosage to improve levels to at least two-thirds of the RDA [20]. As iron deficiency directly affects athletes’ ability to train effectively and calcium deficiency negatively impacts their capacity to remain free from injury, both are appropriate for supplementation [48]. 

### 4.8. Beverages

According to Holtzman’s hierarchy of nutritional needs [13], EA and hydration should be prioritized first, followed by tailored macronutrient composition and maximization of micronutrient intake. Therefore, dancers should prioritize increasing EI and diet variety prior to adopting a supplementation regime, as this facilitates meeting carbohydrate, protein, and micronutrient requirements. One study indicated that pre-professional ballet dancers had poor fluid intake during the week (1768 mL/day) and consumed significantly less on the weekend (1350 mL/day) [22]. Dehydration has been linked to nausea, decreased coordination, muscle cramps, and soreness [8] therefore, hydration is a key factor to be addressed for optimal performance and health. Poor hydration can be attributed to minimal rest breaks and higher fluid requirements, with fluid losses reaching 2 L/h during extended rehearsals [8]. Increased alcohol consumption on weekends may also displace water intake, affect hydration status due to diuretic effects, and exacerbate delayed recovery [49]. If improvements in dietary and fluid intake are unable to resolve nutritional deficiencies, professional guidance is recommended for appropriate supplementation advice and to minimize adverse nutrient interactions or other risks [48]. Sports dietitians can have profound impacts here, using their expertise in sports nutrition principles and understanding of the nuances of dance to effectively guide dancers to address deficiencies and optimize nutrient and hydration status. 

### 4.9. Nutrition Knowledge and Education

Scores on the General Nutrition Knowledge Questionnaire have been negatively correlated with those of the Eating Attitudes Test in professional ballet dancers [50]. Nutrition education may address the misconception that nutrition does not affect performance [21] and educate dancers on the benefits of adequate nutrition on health and performance. The positive effects of nutrition education interventions have also been identified in younger dancers, with contemporary and jazz students showing improvements in nutrition knowledge and eating disorder risk following three workshops focusing on mental health, RED-S, performance effects, nutrition, and recovery strategies [51]. Programs targeting pre-professional dancers have been shown to maintain reductions in cognitive restraint and dieting at 6 months post-education intervention [52]. This has also been replicated in dancers as young as 13 years of age, with increased nutrition knowledge, self-efficacy, and perceived susceptibility to RED-S observed immediately after a three-part video lecture series [53]. Although a decline in nutrition knowledge was observed at follow-up 6 weeks post-intervention, some dietary changes persisted, including a reduction in fast food intake and increased milk consumption. Hence, nutrition education can improve dietary adequacy and act as an early intervention strategy against disordered eating behaviors but needs to be a reiterative process to maintain benefits. Dietitian-led education programs can ensure accurate, evidence-based information is delivered to support dancers, instead of dancers turning to other athletes, friends, parents, and teen magazines for advice [21]. While they may be well-intentioned, they are often misinformed from their own experiences of weight-centric ideals, body discrimination, and disordered eating behaviors, further perpetuating harmful nutrition information to dancers. This risk is highlighted by a previous study on 144 college dance educators, where 73% reported using their personal experience to learn about nutrition and only 19% taking a nutrition course [54]. A shift away from diet culture and the thin ideal in dance will require education of teachers and industry professionals, ensuring organizations and policies are aligned with protecting the physical and mental health of dancers. Education programs should be embedded within dance training at the pre-professional and professional levels, and access to nutrition professionals should be available to athletes in the studio and performance environments. Collaboration with accredited health professionals in these programs, as well as individualized nutrition interventions, can mitigate the risks of developing eating disorders and nutritional deficiencies from lack of education and minimizing harm of subsequent symptomology of LEA and RED-S. 

### 4.10. Strengths/Limitations

This review is the first to scope the literature on dancers’ energy status and diets to assess overall adequacy of their intake to meet energy demands. It employed a comprehensive approach to database selection, including performing arts databases and grey literature, with minimal restrictions. To understand the complexity of nutrition for dancers, there was extensive data extraction for energy, nutrients, and beverages, as well as other important contextual factors, including eating patterns, nutrition knowledge, and supplement use. All but one paper included in this review reported on ballet dancers, further highlighting gaps in the literature and the need for further research into different genres such as musical theatre and opera, the complexities that define them, and the unique requirements presented in each of these styles of dance. 

However, there are also notable limitations to consider. As a scoping review was selected to scope and synthesize the evidence, we are unable to draw implications or recommendations for practice [55]. Only twelve papers were eligible due to the strict definition for pre-professional dancers and limited research into the artform. With ballet as the predominant genre included in this review, the inferences and recommendations made are primarily representative of trends and interventions found in this specific genre and may not be generalizable to dance as a whole sport. Samples were also predominately female, with male subjects underrepresented or excluded from the studies as there were “few” [23]. Five out of twelve included studies were published before the year 2000, and thus may not be reflective of how the dance industry has evolved over time, the growing influence of social media on body image and food knowledge [56], and other sociocultural changes. Data heterogeneity posed a challenge when comparing datasets and referring to sports nutrition recommendations, with only two studies reporting macronutrients by grams per kilogram of bodyweight per day. The validity of the data may have been undermined by recall bias or measurement error. Most of the dietary assessment methods reported rely on memory and self-report and no studies reported the use of the Goldberg method, or any other, to exclude misreporting. Although dual energy x-ray absorptiometry is considered the criterion standard, it is not always accessible or feasible [57]. The field methods of skins-folds are the least affected by day-to-day variation and restrictive and/or compensatory behaviors [57,58]. In fact, the measurement of body composition has potential to cause harm in athletes and should only be done if justified [59]. 

## 5. Conclusions

This review highlights an evidence gap and the need for dietetic intervention within the performing arts industry to support dancers in optimizing their nutrition for health and performance. This review indicated that pre-professional and professional dancers are consuming inadequate energy and macro- and micronutrients compared to their requirements, likely due to the complex interaction of the nuances of the physical demands of the sport and the impact of the perception or expectation for “thinness” on body image. Involvement of accredited sports dietitians in dance groups or for individual support where appropriate may help to increase nutrition knowledge and go some way to assist dance athletes in avoiding problematic LEA, RED-S, and eating disorders and their consequences. Quantification and assessment of dancers’ dietary intake, energy expenditure, and demands of each dance genre will help to inform specific sports nutrition recommendations in this sport.

## Supplementary Materials

The following supporting information can be downloaded at: https://www.mdpi.com/article/10.3390/nu16244293/s1, Table S1. MEDLINE Search Strategy.
