# Supplementary material for: What Do We Know About the Energy Status and Diets of Pre-Professional and Professional Dancers: A Scoping Review
Source: Nutrients. 2024 Dec 12;16(24):4293. doi: 10.3390/nu16244293 (PMC11676647; doi:10.3390/nu16244293)
Supplement: Supplementary file 1 [file nutrients-16-04293-s001.zip › nutrients-3333643-supplementary.pdf]

Table S1. MEDLINE Search Strategy.

Search	Query
**1. Population**	1. Dancing/<br>2. Danc*<br>3. Ballet<br>4. Ballerina<br>5. Musical theatre<br>6. Opera<br>7. or/1-6
**2. Diet**	8. exp Diet/<br>9. exp Food/<br>10. Diet Records/<br>11. Nutrition Surveys/<br>12. Diet Surveys/<br>13. exp “Feeding and Eating Disorders”/<br>14. Nutri*<br>15. Food*<br>16. Diet*<br>17. Meal*<br>18. Eating adj (pattern* or habit* or behavio?r or disorder*)<br>19. Vegetable*<br>20. Legume*<br>21. Bean*

	22. Grain*<br>23. Cereal*<br>24. Wholegrain*<br>25. Fruit*<br>26. Lean Meat*<br>27. Poultry<br>28. Fish*<br>29. Seafood<br>30. Sea food<br>31. Egg*<br>32. Tofu<br>33. Nut<br>34. Nuts<br>35. Seed*<br>36. Dairy<br>37. Milk<br>38. Yog?urt<br>39. Cheese<br>40. Snack*<br>41. Sugar*<br>42. Sweet*<br>43. Dietary Supplements/<br>44. Supplement* adj (multivitamin or vitamin* or mineral* or food or diet*)

	45. Nutraceutical*<br>46. exp Energy intake/<br>47. Calori*<br>48. Megajoule*<br>49. Kilojoule*<br>50. Joule*<br>51. Dens* adj (energy or nutrient)<br>52. Energy adj3 (avaib* or deficien* or deficit* or expend* or balance)<br>53. kcal*<br>54. Kj<br>55. Nutritional status/<br>56. M?cronutrient*<br>57. Protein*<br>58. Fat*<br>59. Carb or carbs or carbohydrate*<br>60. CHO<br>61. Alcohol*<br>62. Fibre<br>63. Fiber<br>64. Iron<br>65. Vit* D<br>66. Calcium<br>67. Vit* B12

	68. Exp Beverages/<br>69. SSB<br>70. Flavo?red adj2 (dairy* or milk* or drink*)<br>71. Drink* adj2 (sugar* or fizzy or carbonated or sweet*)<br>72. Beverage* adj2 (sugar* or fizzy or carbonates or sweet*)<br>73. Soda<br>74. Pepsi<br>75. Cola<br>76. Coke<br>77. Lemonade<br>78. or/8-77<br>79. 7 AND 78
